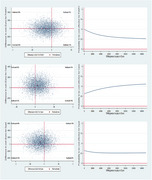# Economic Evaluation of the Digital Application “Support, Monitoring and Reminder Technology for Mild Dementia” for People with Mild Cognitive Impairment and their Informal Caregivers

**DOI:** 10.1002/alz70860_102860

**Published:** 2025-12-23

**Authors:** Zartashia Ghani, Johan Jarl, Peter Anderberg, Johan Sanmartin Berglund, Maria Quintana Aparicio, Pilar Barnestein‐Fonseca, Selim Cellek, Fermín Mayoral Cleries, M Garoellda, Gloria Guerrero‐Pertiñez, Karen Hayden, Carmel Moore, Jufen Zhang, Dominic Trepel, Sanjib Saha

**Affiliations:** ^1^ Applied Health Technology, Department of Health, Blekinge Institute of Technology, Karlskrona, Karlskrona, Sweden; ^2^ Lund University, Lund, Lund, Sweden; ^3^ Department of Health, Blekinge Institute of Technology, Karlskrona, Karlskrona, Sweden; ^4^ Department of Health, Blekinge Institute of Technology (BTH), Karlskrona, Karlskrona, Sweden; ^5^ Brain, Cognition and Behavior, Clinical Research. Consorci Sanitari de Terrassa‐ Hospital Universitari, Barcelona, Barcelona, Sweden; ^6^ , Instituto CUDECA de Estudios e Investigación en Cuidados Paliativos, Fundación CUDECA, Av del Cosmos, Málaga, Malaga, Spain; ^7^ Medical Technology Research Centre, Anglia Ruskin University, ARU Peterborough, University House, Bishop's Road, Peterborough, Peterborough, Peterborough, United Kingdom; ^8^ Hospital Regional Universitario de Málaga U.G.C de Salud Mental, Malaga, Malaga, Spain; ^9^ Consorci Sanitari Terressa, Terrassa, Barcelona, Spain; ^10^ Anglia Ruskin Clinical Trials Unit, School of Medicine, Faculty of Health, Education Medicine and Social Care, Chelmsford, Chelmsford, United Kingdom; ^11^ Anglia Ruskin Clinical Trials Unit, School of Medicine, Faculty of Health, Education Medicine and Social Care, Anglia Ruskin University, Bishops Hall Lane, Chelmsford, Chelmsford, Chelmsford, United Kingdom; ^12^ School of Medicine and Global Brain Health Institute, Trinity College Dublin, Dublin, Ireland; ^13^ Trinity College Dublin, Dublin, Ireland; ^14^ Global Brain Health Institute, Dublin, Ireland

## Abstract

**Background:**

A randomized controlled trial was conducted to evaluate the effectiveness of a tablet‐based application, SMART4MD, in improving or maintaining the quality of life for person with mild cognitive impairment (PwMCI) and their informal caregivers. The objective is to conduct an 18‐month economic evaluation of the SMART4MD app, in addition to standard care, compared to standard care alone in Sweden and Spain from a healthcare provider perspective.

**Method:**

A total of 345 dyads (173 received intervention; 172 received standard care) participated from Sweden, and 347 PwMCI (174 received intervention; 173 received standard care) participated from Spain. The primary outcome measure was quality‐adjusted life years (QALYs). The findings are reported as incremental cost‐effectiveness ratios (ICER).

**Result:**

The intervention was dominated by standard care at the Swedish site, but at the Spanish site, the ICER was €3,337/QALY for PwMCI. For the informal caregivers at the Swedish site, the ICER was €78,000/QALY. However, neither costs nor QALYs were statistically significantly different for dyads or PwMCI in both sites.

**Conclusion:**

The difference in site‐specific results warrants further exploration of the use of the SMART4MD application for PwMCI in terms of cost‐effectiveness.